# EPD in 2020: enhanced data visualization and extension to ncRNA promoters

**DOI:** 10.1093/nar/gkz1014

**Published:** 2019-11-04

**Authors:** Patrick Meylan, René Dreos, Giovanna Ambrosini, Romain Groux, Philipp Bucher

**Affiliations:** 1 Swiss Institute of Bioinformatics (SIB), CH-1015 Lausanne, Switzerland; 2 School of Life Sciences, Swiss Federal Institute of Technology, CH-1015 Lausanne, Switzerland

## Abstract

The Eukaryotic Promoter Database (EPD), available online at https://epd.epfl.ch, provides accurate transcription start site (TSS) information for promoters of 15 model organisms plus corresponding functional genomics data that can be viewed in a genome browser, queried or analyzed via web interfaces, or exported in standard formats (FASTA, BED, CSV) for subsequent analysis with other tools. Recent work has focused on the improvement of the EPD promoter viewers, which use the UCSC Genome Browser as visualization platform. Thousands of high-resolution tracks for CAGE, ChIP-seq and similar data have been generated and organized into public track hubs. Customized, reproducible promoter views, combining EPD-supplied tracks with native UCSC Genome Browser tracks, can be accessed from the organism summary pages or from individual promoter entries. Moreover, thanks to recent improvements and stabilization of ncRNA gene catalogs, we were able to release promoter collections for certain classes of ncRNAs from human and mouse. Furthermore, we developed automatic computational protocols to assign orphan TSS peaks to downstream genes based on paired-end (RAMPAGE) TSS mapping data, which enabled us to add nearly 9000 new entries to the human promoter collection. Since our last article in this journal, EPD was extended to five more model organisms: rhesus monkey, rat, dog, chicken and *Plasmodium falciparum*.

## INTRODUCTION

The Eukaryotic Promoter Database (EPD) was created in 1986 and first published as a table in a journal article (1). Distribution in machine-readable form followed a year later. Promoters are conceptually and operationally defined as transcription start sites (TSS) or transcription initiation regions. The primary goal of EPD has always been to provide accurate TSS annotation based on all experimental evidence available at a given time to computational and bench biologists.

Until about 10 years ago, EPD was a manually curated database derived from experiments published in journal articles. Further to the advent of next-generation sequencing-based whole-genome TSS mapping protocols, in particular CAGE (2), we felt obliged to completely revise our data acquisition and quality control (QC) procedures. Today, we produce comprehensive, organism-specific promoter collections in a completely automatic fashion from high-throughput transcript mapping data and high-quality gene annotation resources. For instance, the current human promoter collection was derived from about 39 trillion (!) sequenced mRNA 5′ ends from ENCODE (3) and FANTOM5 (4), using GENCODE (5) as gene annotation resource. The new organism-specific TSS promoter collections obtained in this way are distributed under the name ‘EPDnew’ to make users aware of the recent changes in data collection procedures.

Manual and semi-automatic QC procedures are still in place, but they are now mostly applied to the incoming experimental data, or to the preliminary results obtained with a new promoter definition pipeline under development. A general description of the production process and QC methods can be found in (6,7). Organism-specific information about source data and computational methods is posted on the EPD summary web pages for the corresponding organisms (e.g. https://epd.epfl.ch/human/human_database.php).

EPD can be queried in a gene-centric or feature-centric way. In the first case, a user interested in a specific gene can retrieve all associated promoters, each having an entry page containing general information, a genome browser screenshot showing the promoter in its genomic context, links to alternative genome browser views, and tools to search for motifs, to retrieve DNA sequences or to explore tissue-specific TSS usage. Alternatively, EPD can be accessed via associated tools developed by our group: the Signal Search Analysis (SSA) (8) and ChIP-Seq (9) web servers. The former enables users to query and select promoters by sequence motif content, and the latter by experimental criteria such as histone modification levels or chromatin accessibility. More on these tools and usage examples can be found in (10). All experimental data accessible via the ChIP-Seq server are stored in the Mass Genome Annotation (MGA) data repository (11) and can be downloaded from there in a standardized format. Furthermore, chromatin profiles (ChIP-seq, DNase-seq, etc.) for selected promoter regions can be extracted in a numerical table format with the previously described ChIP-Extract tool (7).

There are other TSS annotation databases, especially for human and mouse, including refTSS (12) and DBTSS/DBKERO (13). Compared to these resources, EPD is more selective, containing fewer promoters, which on average have stronger support from experimental data. Note also that EPD enforces a minimal distance between two dominant TSSs of 100 bp, which reduces redundancy at a very local scale. This is a convenient property for certain types of computational analysis, for instance when genomic features (DNA motifs, ChIP-seq reads) are counted in windows of similar size.

## RECENT DEVELOPMENTS

### Complete redesign of the UCSC Genome Browser-based promoter viewers

Among the most important advances since our last publication in 2017 is the complete re-implementation of the EPD viewer hub at UCSC, exploiting new data transfer mechanisms that allow for rapid upload of hundreds of data tracks for a specific genomic region of moderate size.

EPD’s visualization tools primarily serve researchers who are interested in a specific promoter and would like to explore the corresponding genomic context in an interactive way. A biologist exploring a promoter region may have the following types of questions in mind: Does the promoter have an open chromatin conformation in a particular cell type? What transcription factors bind to it and what kind of histone marks are present? Are there SNPs with known phenotypes in the vicinity of the promoter? It is important to recognize that for human, mouse and major model organisms, answers to many of such questions are already present in public data. However, efficient tools for finding, accessing, integrating and displaying such information from different sources are lacking. The EPD promoter viewers aim at filling this gap.

Since chromosomes are continuous linear structures with no obvious punctuation marks, a genome browser, enabling users to zoom in and out and move along chromosomes, seems to be the appropriate visualization tool. For the same reason, track hubs are appropriate data structures to organize and represent quantitative genomic information that is collinear with chromosomes. We opted for an implementation that remotely uses the UCSC Genome Browser as visualization platform, mostly because this enables users to view data tracks from EPD jointly with data from the UCSC Genome Browser database (14) and other public track hubs. Of particular significance is the access to epigenetic and gene expression data from major consortia such as ENCODE (3), RoadMap (15), Blueprint (16) and GTEx (17), all available through public track hubs. A UCSC-based implementation offers of course additional advantages, including intuitive and customizable display tools for diverse genomic features such as gene models, chromatin loops, sequence alignments or haplotype groups, or the newly introduced mechanisms for regrouping and clustering of user-selected track sets. Note that refTSS also uses a UCSC Genome Browser track hub for data visualization, whereas other TSS resources (DBTSS, FANTOM5) have developed their own genome browsers for this purpose. Direct navigation links to these third-party viewers are present on the organism summary pages of EPD, as well as on individual promoter entry pages.

The EPD visualization tools comprise two components: track hubs and data viewers. The track hubs contain promoter-relevant data, which are not available from UCSC-resident or public track hubs at the desirable resolution. The content varies a lot between species. For genomes not supported by UCSC, the EPD track hub also provides the genome sequence and gene annotation tracks. For all species, single-base resolution TSS-mapping tracks are provided, in most cases derived from CAGE data. High priority is also given to ChIP-seq profiles for promoter-specific histone marks and RNA polymerase 2, nucleosome maps generated with MNase-seq, and open chromatin profiles obtained with DNase-seq, ATAC-seq or similar assays. Note that the source data of all EPD tracks are available from the MGA repository and thus accessible for promoter selection and analysis via the ChIP-Seq and SSA servers. For some species (including human), track hubs are provided for multiple assemblies, in which case they may contain different track subsets. A detailed description of EPD tracks for the human genome assembly hg38 is given in Supplementary Table S1. Summary statistics for all species can be found in Supplementary Table S2.

EPD data viewers provide stable, promoter-centric views of genomic regions. They are implemented by means of so-called session files, a text file format used by the UCSC Genome Browser that defines the subset of data tracks to be displayed, the display mode for each track and the order in which they should appear in the browser window. Users can access the promoter viewers through hyperlinks from the organism summary pages or from individual promoter entry pages. In the latter case, they will be directly taken to the corresponding promoter regions. Session links are provided for both the genome browser at UCSC and its European mirror.

**Figure 1.**
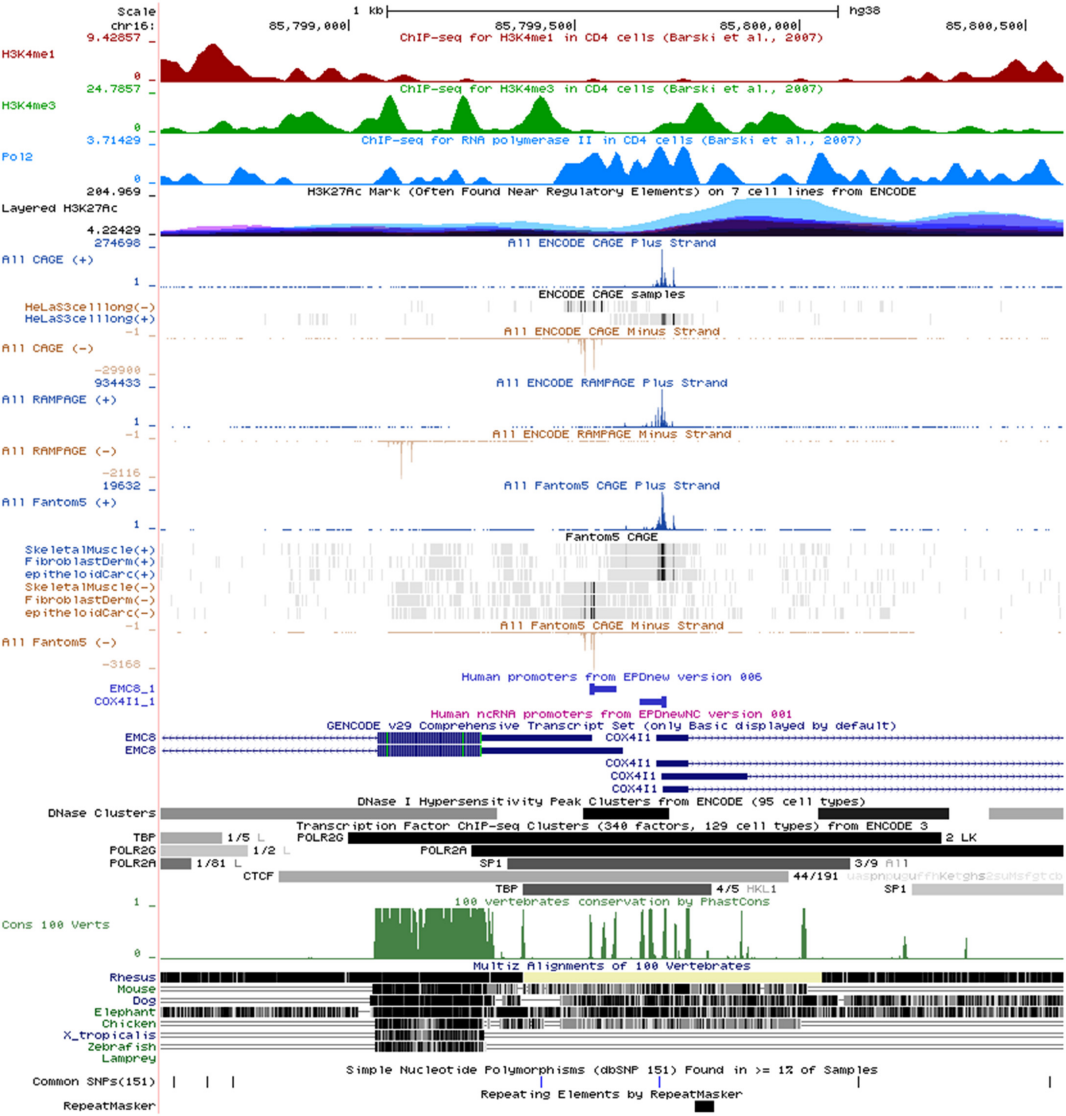
Screen shot of a UCSC Genome Browser window showing two divergent human promoters for genes EMC8 and COX4l1, as displayed by the EPD viewer. From top to bottom (track source in parentheses): high-resolution ChIP-seq tracks for histone modifications H3K4me1, H3K4me3 and RNA polymerase 2 (EPD); lower resolution ChIP-seq tracks for H3K27ac (ENCODE-UCSC); single base resolution CAGE tracks for ENCODE, RAMPAGE and FANTOM5, all libraries combined plus a few selected tissue-specific libraries (EPD); EPD promoter annotation track, thin and thick lines mark 50 bp upstream and10 bp downstream regions relative to the dominant TSS (EPD); GENCODE genes (UCSC); open chromatin/DNase I clusters (ENCODE-UCSC); ChIP-seq peaks for selected transcription factors and RNA polymerase 2 subunits (ENCODE-UCSC); sequence conservation tracks, common SNPs from dbSNP and repetitive elements from RepeatMasker (UCSC).

EPD promoter viewers are intended to be useful starting points, not endpoints for exploring a genomic region. From there, researchers are expected to zoom in or out on specific regions, to reconfigure the display in various ways, to add new tracks or to incorporate their own data in the view. An example of a promoter view is shown in Figure 1. In general, we try to keep the initial view as compact as possible such that it fits into the display area of a computer monitor. We try to present an optimal selection of tracks tailored to the interests of promoter researchers. The tracks come from three types of sources: the UCSC Genome Browser database, the EPD track hub or public track hubs registered at UCSC. Currently, all EPD-provided track sets are represented by at least one track, thereby enabling users to rapidly find and load additional tracks from the same set by right-clicking on the track set name displayed on the left-hand side of the browser window.

In addition to the generic viewers that are available for all 15 organisms covered by EPD, we have started to provide specialized viewers for specific cell types or tissues. For instance, we offer a specific viewer for the lymphoblastoid cell line GM12878, which has been extensively assayed by the ENCODE consortium (Supplementary Figure S1).

Very recently, we introduced promoter-specific views for human and mouse promoters, motivated by the observation that the TSS signal of certain highly tissue-specific promoters was barely perceptible in the FANTOM5 or ENCODE CAGE tracks derived from all libraries combined. These viewers are variants of the standard viewer, in which the combined CAGE tracks are replaced by the three tissue-specific CAGE tracks displaying the highest expression levels for the corresponding promoters.

### Extension of EPDnew to ncRNA promoters

EPD relies on external resources for gene annotation, which must be reasonably stable over time and enjoy broad acceptance by the research community. Until a few years ago, such resources were lacking for ncRNA genes. However, GENCODE (5) has recently made considerable efforts to fill this gap and is now offering quality-controlled gene annotations for all major classes of ncRNAs from human and mouse. The corresponding gene nomenclature committees, HGNC and MGNC (18), have followed suit by assigning official gene symbols to many of these newly annotated genes. We thus felt that the time has come to include ncRNA promoters in EPD.

**Figure 2.**
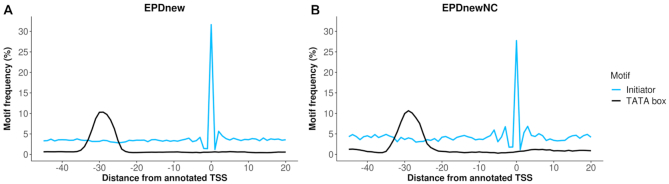
Frequency and positional distribution of core promoter motifs relative to the TSS of human coding and noncoding genes as defined in EPD.

Our pipelines for inferring consensus TSS positions from high-throughput data are optimized for protein-coding genes. The same holds for currently used high-throughput technologies for genome-wide TSS mapping. It was thus not obvious whether the established procedures of EPD would work for ncRNAs. It is important to recognize that ncRNAs are a heterogeneous group of molecules, transcribed by different polymerases, undergoing diverse post-transcriptional processing events and localized in different subcellular compartments. In view of the expected obstacles and uncertainties, we decided to test our standard TSS inference procedures separately on different classes of ncRNAs, using the ‘biotype’ annotation by GENCODE as classification criterion. As with mRNA promoters, we used motif enrichment as a proxy for TSS mapping accuracy. Using stringent quality control criteria, we were able to generate high-quality promoter collections for the ‘antisense’ and ‘long intergenic’ biotypes, and consequently incorporated promoters for these two classes into the publicly released ncRNA promoter collections. In total, we accepted 2339 ncRNA promoter from human and 3077 from mouse. Note that the frequencies and positional distributions of the TATA-box and initiator motifs are almost identical between the human coding and noncoding promoters, suggesting similar TSS mapping accuracy of the two independently generated collections (Figure 2). An example of a promoter view of an antisense ncRNA can be found in Supplementary Figure S2.

### Leveraging RAMPAGE data

RAMPAGE (19) is a TSS mapping technique that uses paired-end sequencing to uniquely assign a TSS defined by the 5′ read to an annotated gene matched by the 3′ read of the same pair. High-coverage RAMPAGE data have been publicly released for >200 human tissues. We used these data to assign previously ‘orphan’ TSS peaks to genes. (Here ‘orphan’ refers to peaks that could not be assigned to genes via our proximity criterion requiring that they fall within 100 bp from a transcript start annotated in GENCODE.)

The specific nature of this data type required modifications of the standard promoter inference procedures. First, we filtered the RAMPAGE data by keeping only read pairs where we were able to map the 3′ read to a known protein-coding gene. For each such read pair, we then kept the genomic position of the 5′ end and the gene symbol provided by the 3′ read. The 5′ reads were clustered in the same way as we do for traditional CAGE data. However, the mapping of the TSS peaks to genes was done on the basis of the gene symbols associated with the individual reads rather than by proximity to annotated transcript starts.

Standard EPD quality controls using motif distributions suggested that the RAMPAGE-inferred TSS positions were not as precise as those obtained from CAGE. We thus used the RAMPAGE data only for mapping CAGE peaks to genes but not for choosing the reference TSS position in the promoter database. Specifically, we used RAMPAGE-inferred TSS positions as new transcription start annotations to associate orphan CAGE peaks with genes via the same proximity criterion that we use for GENCODE gene annotations. In total, this enabled us to validate and add 8806 additional promoters to the current human promoter collection (human EPDnew version 6).

### Additional developments since January 2017

New promoter collections were released for chicken, dog, rat, rhesus monkey and the malaria parasite *Plasmodium falciparum*. New versions were released for human, mouse, *Drosophila*, *Arabidopsis* and fission yeast (*Schizosaccharomyces pombe*). The current promoter entry totals are given in Table 1.

**Table 1.** Organisms covered in EPD and corresponding promoter totals (September 2019)

Mammals		Other animals		Other eukaryotes	
*Homo sapiens*	29598 (2339)	*Drosophila melanogaster*	16972	*Arabidopsis thaliana*	22703
*Mus musculus*	25111 (3077)	*Danio rerio*	10728	*Zea mays*	17081
*Rattus norvegicus*	12601	*Caenorhabditis elegans*	7120	*Plasmodium falciparum*	5597
*Macaca mulatta*	9575	*Apis mellifera*	6493	*Saccharomyces cerevisiae*	5113
*Canis lupus familiaris*	7545	*Gallus gallus*	6127	*Schizosaccharomyces pombe*	4802

Numbers in parentheses relate to promoters of noncoding RNAs.

With the release of a *Plasmodium* promoter collection, EPD covers for the first time a human pathogen. This is a significant step forward in a new direction. A genome browser view of a *Plasmodium* promoter is shown in Supplementary Figure S3. Interesting is the nucleosome track, which shows rigidly positioned nucleosomes downstream of the promoter organized in a regularly spaced array, as seen in other species covered by EPD.

## DATA AVAILABILITY

EPDnew is freely accessible without the need for preregistration. Web-based access is provided via the EPD website at https://epd.epfl.ch. Data files can be downloaded in text format via FTP from ftp://ccg.epfl.ch/epdnew/.

## Supplementary Material

gkz1014_Supplemental_File

## Notes

Present address: René Dreos, Center for Integrative Genomics, University of Lausanne, CH-1015 Lausanne, Switzerland.
