# Supplementary material for: EPD in 2020: enhanced data visualization and extension to ncRNA promoters
Source: Nucleic Acids Res. 2019 Nov 4;48(D1):D65–9. doi: 10.1093/nar/gkz1014 (PMC7145694; doi:10.1093/nar/gkz1014)
Supplement: gkz1014_Supplemental_File [file gkz1014_supplemental_file.pdf]

# EPD in 2020: Enhanced data visualization and extension to ncRNA promoters. Supplementary material.

Patrick Meylan[1], René Dreos[1], Giovanna Ambrosini[1,2], Romain Groux[1,2] and Philipp Bucher[1,2,*]

[1] Swiss Institute of Bioinformatics (SIB), Lausanne, CH-1015, Switzerland
[2] School of Life Sciences, Swiss Federal Institute of Technology, Lausanne, CH-1015, Switzerland

*To whom correspondence should be addressed. Tel: +41 21 693 0956; Fax: +41 21 693 1850; Email: philipp.bucher@epfl.ch.

Present Address: René Dreos, Center for Integrative Genomics, University of Lausanne, Lausanne, CH-1015, Switzerland

## Supplementary Tables:

**Table S1.** Content of the generic EPD viewer for human genome assembly hg38

| UCSC track name | Description | 1 bp[a] | 20 bp[a] | 100 bp[a] | Format | Reference |
|---|---|---|---|---|---|---|
| Selected histone marks | H3K4me1 and H3K4me3 ChIP-seq tags | - | 2 | - | wig | Barski 2007 |
| Pol2 | RNA polymerase II ChIP-seq tags | - | 1 | - | wig | Barski 2007 |
| Roadmap histones | Roadmap histone marks, 116 tissues | - | 434 | 347 | wig | Romanoski 2015 |
| All CAGE (+) | 5' end of ENCODE CAGE reads, cell lines merged | 1 | - | - | wig | Djebali 2012 |
| ENCODE CAGE | 5' end of ENCODE CAGE reads, 35 cell lines | 70[b] | - | - | wig | Djebali 2012 |
| All CAGE (-) | 5' end of ENCODE CAGE reads, all cell lines merged | 1 | - | - | wig | Djebali 2012 |
| All RAMPAGE (+) | 5' end of RAMPAGE reads, all tissues merged | 1 | - | - | wig | Batut 2016 |
| All RAMPAGE (-) | 5' end of RAMPAGE reads, all tissues merged | 1 | - | - | wig | Batut 2016 |
| All Fantom5 (+) | 5' end of FANTOM5 CAGE reads, all tissues merged | 1 | - | - | wig | Forrest 2014 |
| Fantom5 CAGE | 5' end of FANTOM5 CAGE reads, 555 tissues | 1110[b] | - | - | wig | Forrest 2014 |
| All Fantom5 (-) | 5' end of FANTOM5 CAGE reads, all tissues merged | 1 | - | - | wig | Forrest 2014 |
| EPDnew promoters | EPDnew promoters version 006 | 1 | - | - | bed | Dreos 2017 |
| EPDnewNC promoters | EPDnew non-coding promoters version 001 | 1 | - | - | bed | Meylan 2020 |
| [a] wig file resolution, [b]plus and minus strands are on separate tracks | | | | | | |

**References:**

Barski et al. 2007. High-resolution profiling of histone methylations in the human genome. Cell 129, 823-837.

Romanoski et al. 2015. Epigenomics: Roadmap for regulation. Nature 518, 314-316.

Djebali et al. 2012. Landscape of transcription in human cells. Nature 489, 101-108.

Batut et al. 2016. High-fidelity promoter profiling reveals widespread alternative promoter usage and transposon-driven developmental gene expression. Genome Res. 23, 169-180.

Forrest et al. 2014. A promoter-level mammalian expression atlas. Nature 507, 462-470

Dreos et al. 2017. The eukaryotic promoter database in its 30th year: focus on non-vertebrate organisms. Nucleic Acids Res. 45, D51-D55.

Meylan et al. 2020. (This paper).

**Table S2.** Contents overview of EPD track hubs

| Organism | Assembly | Number of tracks (resolution in bp) | | | |
|---|---|---|---|---|---|
| | | TSS | ChIP-seq | Nucleosomes | Total |
| *H. sapiens* | *hg38* | 1318 (1) | 3 (20/100[a]) | | 1321 |
| *H. sapiens* | *hg19* | 1176 (1) | 3 (20/100) | | 1179 |
| *M. musculus* | *mm10* | 1018 (1) | 3 (10) | 4 (20) | 1025 |
| *M. mulatta* | *rheMac8* | 32 (1) | | | 32 |
| *D. rerio* | *danRer7* | 26 (1) | 2 (10) | 2 (25) | 30 |
| *P. falciparum* | *pfa2* | 14 (1) | | 8 (10) | 22 |
| *D. melanogaster* | *dm6* | 16 (1) | 2 (10) | | 18 |
| *R. norvegicus* | *rn6* | 12 (1) | | | 12 |
| *C. familiaris* | *canFam3* | 10 (1) | | | 10 |
| *C. elegans* | *ce6* | 2 (1) | 1 (10) | 5 (10) | 8 |
| *S. cerevisiae* | *sacCer3* | 2 (1) | 2 (10) | 1 (10) | 5 |
| *A. thaliana* | *araTha1* | 2 (1) | 1 (20) | 1 (20) | 4 |
| *S. pombe* | *spo2* | 2 (1) | 1 (10) | 1 (20) | 4 |
| *G. gallus* | *galGal5* | 2 (1) | 1 (25) | | 3 |
| *Z. mays* | *zm3* | 2 (1) | 1 (25) | | 3 |
| *A. mellifera* | *amel5* | 2 (1) | | | 2 |

[a]20 bp for histone acetylations and H3K4 methylations, 100 bp for the other marks

## Supplementary Figures:

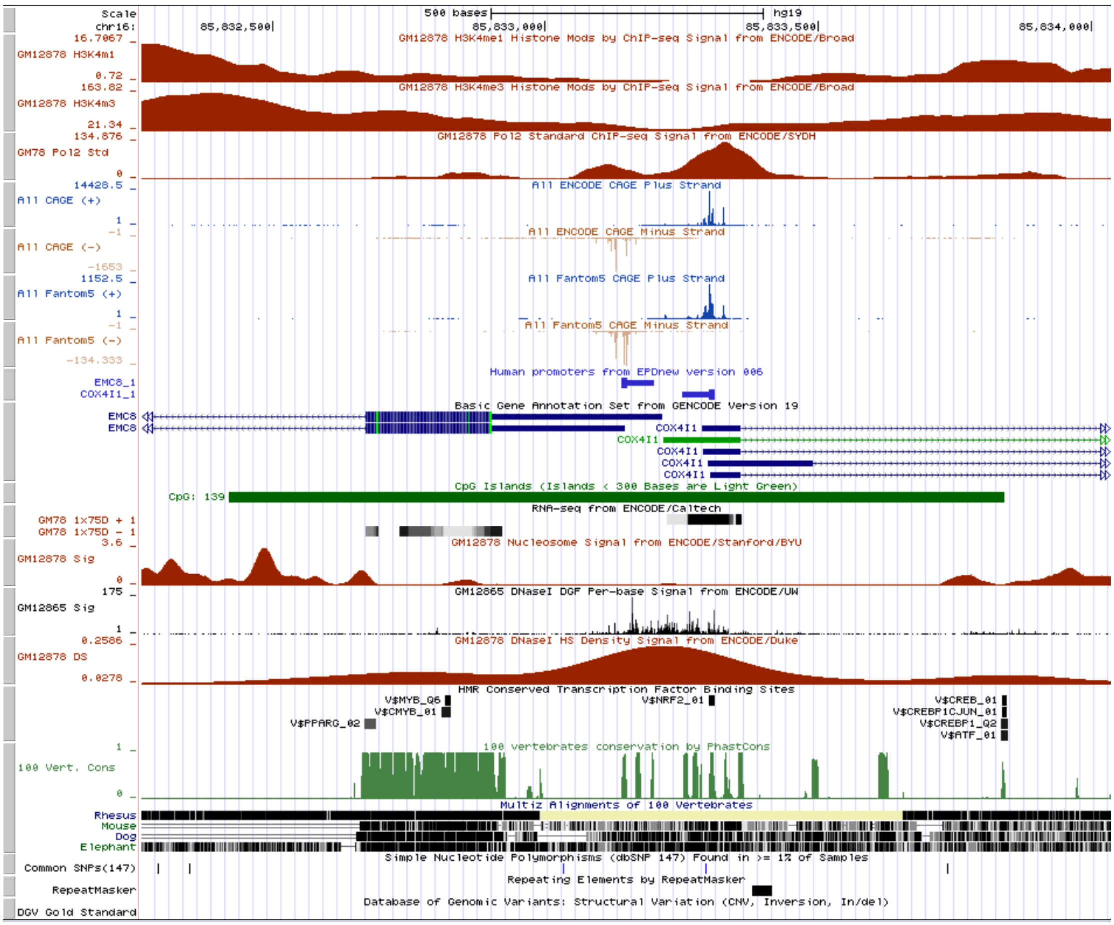


**Figure S1**. Genome browser screenshot of a promoter region visualized with the cell type-specific viewer for lymphoblastoid cell line GM12878. From top two bottom (track source in parentheses): ChIP-seq tracks for histone H3K4me1, H3K4me3 and RNA polymerase 2 (ENCODE-UCSC); single base resolution CAGE tracks for ENCODE and FANTOM5, all libraries combined (EPD); EPD promoter annotation track, thin and thick lines mark 50 bp upstream and10 bp downstream regions relative to the dominant TSS (EPD); GENCODE genes (UCSC); CpG islands (UCSC); Caltech RNA-seq (ENCODE-UCSC); Nucleosomes (ENCODE-UCSC); Single-base resolution digital genomic footprints (ENCODE-UCSC); Low resolution DNase-seq track (ENCODE-UCSC); ChIP-seq peaks for selected transcription factors and RNA polymerase 2 subunits (ENCODE-UCSC); sequence conservation tracks, common SNPs from dbSNP and repetitive elements from RepeatMasker (UCSC). All experimental data tracks relate specifically to cell line GM12878.

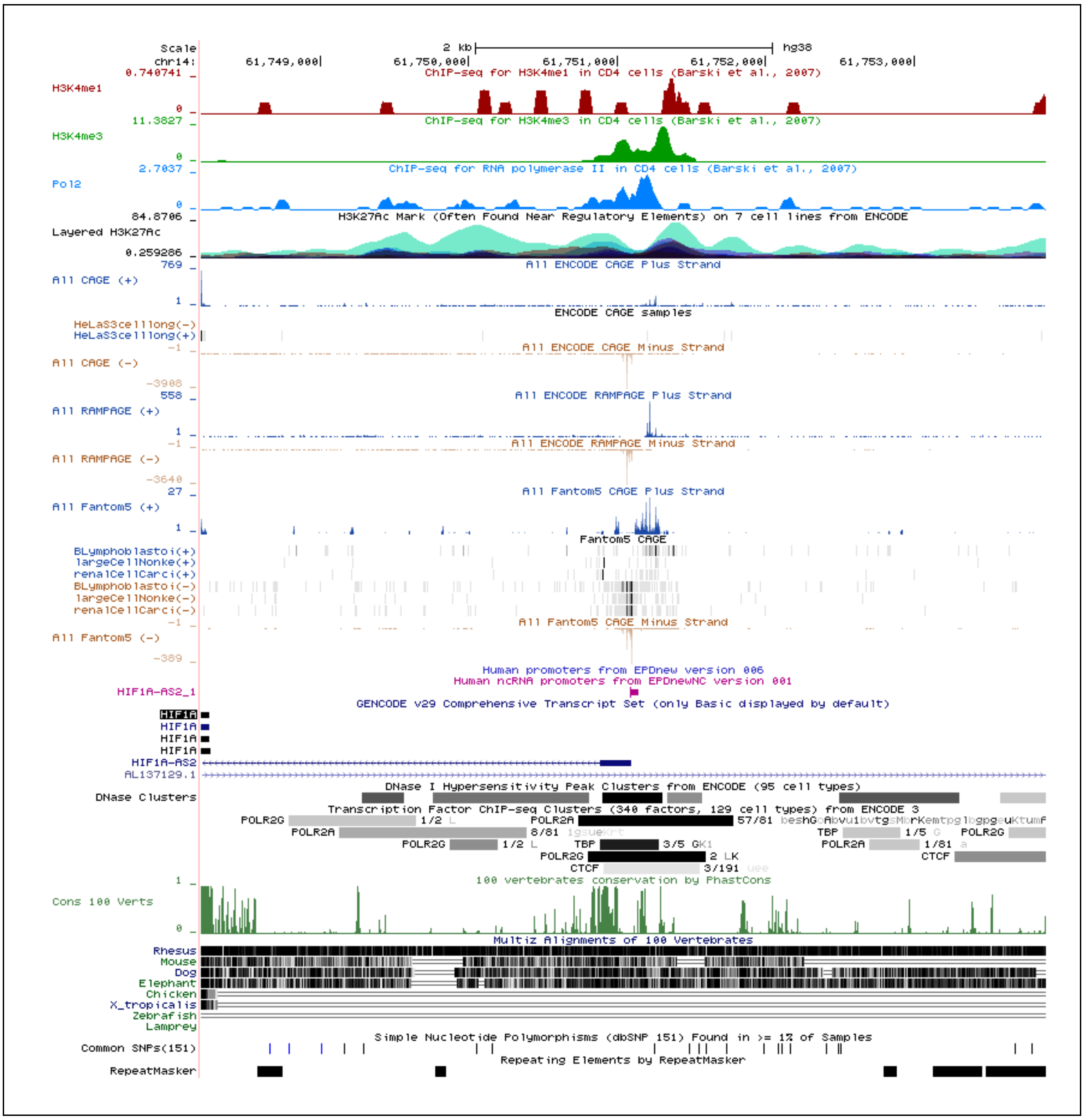


**Figure S2.** Genome browser screenshot of a human promoter of a non-coding RNA (gene HIF1A-AS2).

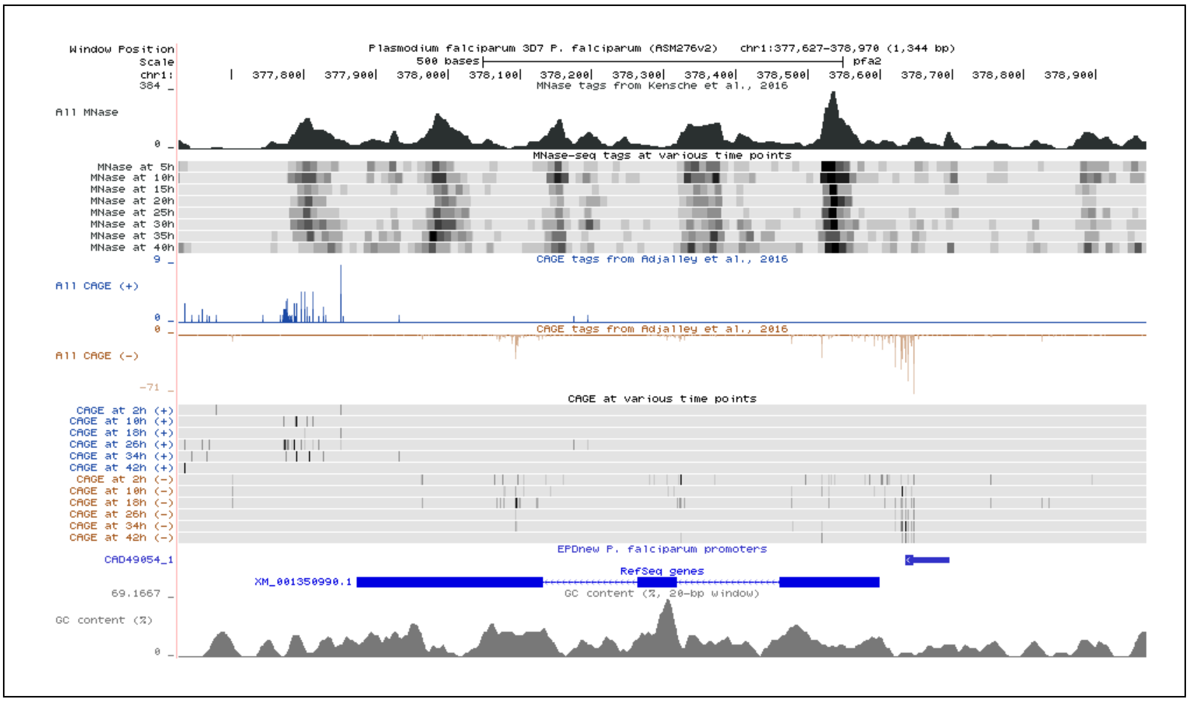


**Figure S3.** Screen shot of the *Plasmodium falciparum* viewer showing a promoter on the minus strand, with MNase tracks showing nucleosome phasing downstream of the TSS. The time points correspond to various stages of the intra-erythrocytic development cycle (IDC). Original data from Kensche et al. 2016, Nucleic Acids Res. 44, 2110–2124.
